# Applications of Transgenic and Knockout Mice in Alcohol Research

**Published:** 2000

**Authors:** Barbara J. Bowers

**Affiliations:** Barbara J. Bowers, Ph.D., is a research associate at the Institute for Behavioral Genetics, University of Colorado, Boulder, Colorado

**Keywords:** animal model, transgenic technology, gene knockout technology, gene expression, DNA, neurotransmitters, GABA receptors, stem cell, receptor proteins, protein kinases

## Abstract

Multiple genetic and environmental factors contribute to the development of alcoholism. Researchers attempting to elucidate the roles of specific genes in alcoholism risk have benefited from advances in genetic engineering. Two important tools used by researchers include transgenic mice, in which a foreign gene is integrated into an animal’s genetic material, and knockout/knock-in mice, in which targeted genes either are rendered nonfunctional or are altered. Both of these animal models are currently used in alcohol research to determine how genes may influence the development of alcoholism in humans.

Alcoholism is a complex disorder that encompasses several physiological and behavioral characteristics (also referred to as phenotypes), including atypical responses to alcohol in initial sensitivity, tolerance, consumption, and withdrawal as well as vulnerability to the rewarding effects of alcohol. Human and animal studies have both shown that these aspects of alcoholism are often mediated by chemical pathways in the brain, known as neurotransmitter systems (Diamond and Gordon 1997; Harris 1999). A major goal for neuroscientists has been to identify genes and proteins in the brain that influence the expression of alcoholism. Increased knowledge of chemicals in the brain and receptor proteins (i.e., protein molecules that recognize and bind neurotransmitters) has provided scientists with a priori reasons for studying specific genes and proteins, which are sometimes referred to as candidate genes and proteins. For example, alcohol affects nearly all brain activity, including enhancement of inhibition, mediated by the gamma-aminobutyric acid (GABA) neurotransmitter system; activation of reward pathways that are dopaminergic (DA) and serotonergic (5–HT); and effects on enzyme proteins that are located inside nerve cells (i.e., neurons) (Diamond and Gordon 1997; Grobin et al. 1998). Therefore, these systems (as well as several others, including nonneuronal genes) have produced many candidate genes to investigate for their roles in the development of alcoholism.

One approach that is particularly effective in the hunt for candidate genes is the reverse genetics approach, in which a gene of interest is altered to change its expression, or function, in the entire animal. This approach involves generating transgenic mice, in which a foreign gene is integrated into an animal’s genetic material, as well as knockout or knock-in mice, in which candidate genes are either inactivated or altered, resulting in a lack of or change in protein expression. Although mouse models may seem poor research tools for studying the genetics of human neurophysiology and behavior, substantial genetic similarities exist between mice and humans, and known correspondence exists between mouse chromosomal regions and human chromosomes (Silver 1995).

This article describes the generation of transgenic mice and knockout/ knock-in mice and recent examples of how these techniques have been applied in alcohol research.

## Transgenics

In the context of mouse models, the term transgenic refers to the introduction of a foreign^1^ gene (known as a transgene) into the genetic material of a mouse in both the reproductive (i.e., germ) cells and the nonreproductive (i.e., somatic) cells. This process leads to the expression and propagation of the gene across future generations. Often the purpose of this technique is to create mice that express more than normal amounts of the gene product (i.e., protein). In some applications, however, the scientific goal is to introduce a different form of the gene in question. This technique allows researchers to evaluate the role of specific genes in the development of disease. For example, a line of transgenic mice generated to study alcoholism would carry a gene that is known or suspected to have a role in some aspect of the disease. Researchers can then study the animals’ behaviors to evaluate the role of the gene in alcoholism.

### Creating Transgenic Mice

Basic requirements for creating a transgenic mouse include (1) identifying and isolating the candidate gene of interest from its original organism (i.e., from the DNA of the organism’s cells) and (2) selecting a suitable promoter that is placed adjacent to the transgene. Promoters are stretches of DNA associated with a specific gene that guide the expression of the gene to specific areas in the brain and turn the expression of the gene “on” either before birth (i.e., prenatally) or after birth (i.e., postnatally). The choice of promoter by the scientist depends on its location in the brain and the time in which (i.e., prenatal or postnatal) the transgene must be expressed. For example, the promoter for the α-calmodulin kinase II (αCamKII) enzyme gene directs postnatal expression to the forebrain. Therefore, any gene associated with this promoter would be found in the forebrain after birth.

**Figure 1:**
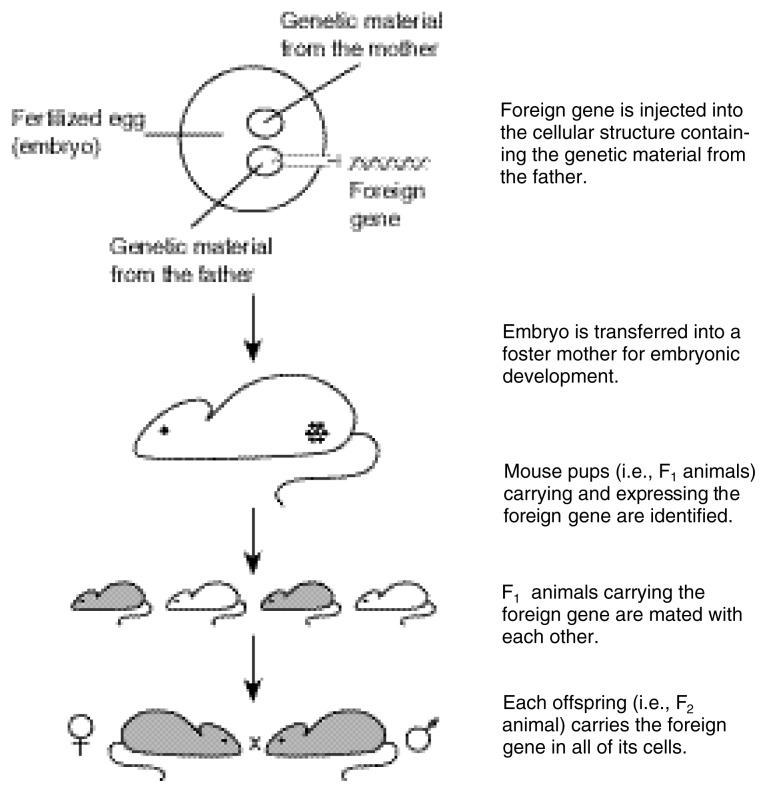
General procedure for the generation of transgenic mice.

Once the transgene construct (i.e., the promoter and DNA) is produced, many copies are introduced into fertilized mouse eggs (i.e., embryos) at the single-cell stage (see figure 1). To create mouse embryos, female mice are hormonally induced to hyperovulate and then mated to males for fertilization. The fertilized eggs are harvested from the female and injected with the transgene. The injection takes place early after fertilization, when the embryo contains two sets of DNA, one from each parent. Each set of DNA exists in a separate structure, called a pronucleus; therefore, both male and female pronuclei exist. Copies of the promoter and foreign gene construct are microinjected directly into the male pronucleus with a fine glass needle. The researcher injects the transgene before the first cell division to ensure that the DNA will develop in all cells of the adult animal. The pronuclei then fuse, and the cell begins to divide normally.

Approximately 50 to 90 percent of the eggs survive the procedure and are implanted into the oviducts of foster mothers, where the embryos develop to term. Mice that carry the transgene, identified using DNA analysis techniques, are called progenitor or founder mice. These mice are bred with non-transgenic mice, and the offspring are tested for the presence of the transgene to confirm that the transgene has integrated into the germ cells. If the integration has occurred, subsequent breeding continues between mice carrying the transgene, referred to as the F_1_ generation, producing lines of transgenic mice (F_2_ generation). For more information on creating transgenic mice, see Camper 1987 and Picciotto and Wickman 1998.

Although researchers can control the expression of the transgene with the choice of promoter, one limitation of the transgenic technique is its inability to target the integration of the transgene to its natural location on the chromosome. The site of integration is unique for each microinjection, and the transgene can be randomly inserted anywhere on any chromosome. This outcome can result in the disruption of a sequence of one of the host animal’s own genes (i.e., known as insertional mutagenesis), producing changes in behavior that could mistakenly be attributed to the transgene itself. Also, the number of integrated copies of the transgene cannot be controlled, and having more copies of a gene does not necessarily indicate increased overexpression of the gene. To control for this, the existence of more than one founder and consequently more than one line of mice for each transgene is desirable. The site of integration and level of expression will differ in each founder, and transgenic mice that descend from the same founder will share the same chromosomal integration site. If each transgenic line displays the same changes in behavior, it is more likely that it is attributable to the transgene.

### Using Transgenic Mice in Alcohol Research

Transgenic mice have traditionally been used to study developmental processes and as models of human diseases. Although transgenic lines have been generated for several genes, their use has been somewhat limited in alcohol research. However, this application remains useful for identifying candidate genes that underlie specific aspects of alcoholism (Wehner and Bowers 1995). For example, animal studies have shown that the serotonergic (5–HT) neurotransmitter system is involved in alcohol consumption and other alcohol-related behaviors (Li and McBride 1995). Therefore, Engel and colleagues (1998) created transgenic mice overexpressing the 5–HT_3_ receptor protein to investigate its role in alcohol and other drug abuse. The researchers used the αCaMKII promoter to direct expression of the 5–HT_3_ transgene to the forebrain. Expression of the 5–HT_3_ receptor was greatly increased in the transgenic mice; receptor binding of a 5–HT_3_ agonist (i.e., a chemical that mimics serotonin at the 5–HT_3_ sub-type) was increased nearly a hundredfold in cortical regions of the brain in one of four transgenic lines tested. The remaining lines exhibited lower levels of receptor expression.

The transgenic mice were tested for alcohol drinking (Engel et al. 1998) and initial sensitivity to alcohol (Engel and Allan 1999). Results indicated that overexpression of 5–HT_3_ receptors decreased alcohol consumption by 46 percent. In support of the role of 5–HT_3_ expression in alcohol consumption, Engel and colleagues (1998) reported that the level of alcohol consumption in the four transgenic lines was related to their levels of receptor overexpression (i.e., the greater the level of over-expression, the greater the reduction in consumption). In contrast, overexpression of 5–HT_3_ receptors increased sensitivity to the activating effects of low doses of alcohol. When some mice are administered low doses of alcohol, their locomotor activity increases. This phenomenon is referred to as alcohol-induced hyperlocomotion and is a measure used in mice to test for initial sensitivity to alcohol. The authors have suggested that 5–HT_3_ receptors play a role in increased initial sensitivity that may be related to decreased alcohol consumption.

The GABA neurotransmitter system provides another avenue for using transgenics in alcohol research. The GABA_A_ receptor family consists of at least 16 different protein molecules (i.e., subunits) assembled 5 at a time, creating a receptor complex that surrounds a channel. When GABA or GABA-like compounds bind to the receptor and activate it, this channel temporarily opens and allows the passage of negatively charged molecules (i.e., chloride ions) to pass from the cell’s exterior to its interior. This ion flow decreases the cell’s excitability, which results in inhibition. The GABA system is the primary modulator of inhibition in the brain (Barnard et al. 1998). Alcohol enhances the inhibitory effects of GABA at the GABA_A_ receptor; this interaction of alcohol and the receptor appears to cause alcohol’s intoxicating and sedating effects.

Numerous studies have established that acute and chronic behavioral effects of alcohol are differentially mediated through GABAergic receptor subunits (for a review, see Grobin et al. 1998). The γ2 subunit, which exists in both a long (γ2L) and short (γ2S) version, has been studied for its role in GABAergic sensitivity to alcohol. Although initial in vitro (i.e., in a test tube) studies investigating the role of the γ2L subunit in alcohol potentiation of GABAergic function showed that this subunit was necessary for alcohol sensitivity; later studies have not found an absolute γ2L requirement (Grobin et al. 1998; Sapp and Yeh 1998). The inconsistencies in these results may be attributable to differences in the in vitro preparations used by the investigators.

To further investigate the function of the γ2 subunit in a whole animal model, Wick and colleagues (2000) created transgenic mouse lines overexpressing the γ2L and γ2S genes. Two γ2L and one γ2S lines of transgenic mice were tested for responses to alcohol, including sedation, ataxia (i.e., loss of coordination), withdrawal seizures, and acute functional tolerance (AFT) (i.e., a measure of tolerance to alcohol that occurs within one testing session as opposed to tolerance development that occurs over several days of alcohol treatment and testing).

None of the transgenic lines of mice displayed altered responses to alcohol, with the exception of AFT, in which tolerance was decreased in both γ2L and γ2S transgenic lines compared with nontransgenic mice. The lack of specificity of the γ2L transgene and lack of effects on the other measures may have been caused by insufficient overexpression of either transgene. However, the levels of expression were sufficient to rescue the lethal phenotype of gene-targeted mutant mice lacking the entire γ2 gene In other words, mice lacking the entire γ2 gene but overexpressing the γ2L transgene survived (Bauer et al. 2000).

The 5–HT_3_ and γ2L and γ2S transgenic lines are two examples of the use of this technology in alcohol research with a focus on initial sensitivity, tolerance, or consumption. Other transgenic mouse lines have been used to study diverse alcohol-related phenotypes, such as alcohol’s effects on aggression (i.e., transforming growth factor α transgenics [TGFα]); alcohol as a cofactor in HIV disease (i.e., transactivator protein overexpression [Tat]); alcohol’s effects on alcohol cardiomyopathy (i.e., alcohol dehydrogenase transgenics [ADH]); and alcohol-induced neurotoxicity in neonatal cerebellum (i.e., cell repressor gene transgenics [bcl-2]) (see table for references).

### Gene-Targeting Techniques in Knockout and Knock-in Mice

Knockout and knock-in mice are created by gene-targeting techniques that produce animals in which a specific gene has been deleted (i.e., “knocked out”) or mutated (i.e., “knocked in”). Ideally, by inference, any differences in phenotype observed in knockout and knock-in mice can be due to the nonfunctional or altered gene. However, adaptations during development attributable to the mutation and the expression of the background genotype may also produce changes in behavior not directly caused by the missing or mutated gene.

In some instances, when a gene vital to embryonic development is deleted or mutated, knockout or knock-in mice lacking the vital gene cannot develop beyond a certain stage, demonstrating the gene’s requirement for developmental processes. In this case, the techniques of transgenics and gene-targeted mutagenesis can be combined to provide added experimental proof that a deleted or mutated candidate gene is actually the gene responsible for the mutated phenotype. This is accomplished by inserting the wild-type gene (i.e., the normal form of the gene) as a transgene into the host genome of the mutant mouse, as in the γ2 knockout mice previously described. If the resulting phenotype is equivalent to the wild-type phenotype, the mutation is considered “rescued,” and presumably the candidate gene is involved in the neurochemical pathway of interest.

### Creating Knockout and Knock-in Mice

To create either knockout or knock-in mice, researchers use targeted mutagenesis, the site-specific (vs. random) integration of a mutated gene using the cell’s natural homologous recombination^2^ mechanism that occurs during DNA replication. In other words, unlike the transgenic techniques, the mutated gene is inserted into its normal location on the chromosome (i.e., the gene is “targeted”). This eliminates the problems associated with insertional mutagenesis seen in some transgenic lines. Gene-targeting traditionally generates knockout mice in which a candidate gene is rendered nonfunctional. Recently this technique has been used to create knock-in mice, in which a mutation is introduced into a candidate gene and the function of the gene is changed but not eliminated.

**Figure 2:**
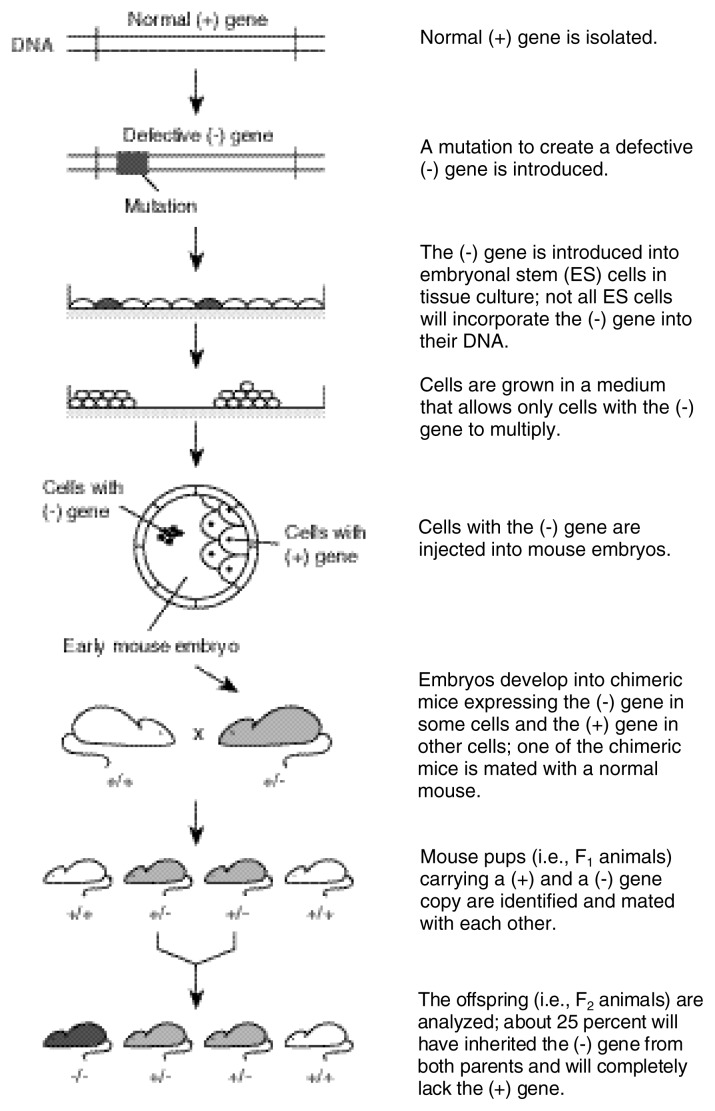
General procedure for the generation of knockout mice.

Requirements of this method include the following: (1) the identification and isolation of the candidate gene from mouse DNA and (2) cultured, mouse embryonic stem (ES) cells. ES cells retain the ability to differentiate into all tissues of a developing mouse. Through genetic engineering techniques, the isolated gene is mutated either to make it nonfunctional or to change its function. The gene is then introduced into the ES cell (see figure 2, p. 182), rather than into an embryo, as in the creation of transgenic mice. Once in the ES cell, the mutated gene changes places with the cell’s normal (wild-type) gene through homologous recombination (for a review, see Capecchi 1994; Picciotto and Wickman 1998). When this occurs, the cell’s wild-type gene is disrupted and becomes nonfunctional (or altered if the knock-in strategy is used). ES cells expressing the mutated gene are identified by growing the cells in a petri dish in a specific medium in which only modified cells can survive. Positive ES cells are microinjected into 3.5 day-old blastocysts (i.e., fertilized embryos consisting of 8 to 16 cells). Researchers implant the blastocysts into foster mothers, where they develop to term. Because the ES cells are introduced at later stages of embryonic cell division, the resulting mouse will be chimeric for the mutated gene (i.e., the mouse will carry the mutated gene in some, but not all, cells.) Further breeding and DNA analysis are required to identify founder mice, which carry the gene in their germ cells. These mice are used to generate lines of knockout mice. This method does not require multiple lines of mice, because the mutation does not insert randomly and only one copy of the mutated gene will have integrated.

Caution is necessary when interpreting results from gene-targeting experiments because of two potentially confounding factors. First, other genes may compensate in response to the disrupted gene, which is nonfunctional throughout prenatal and postnatal development. However, recent advances in gene-targeting techniques that allow researchers to control where and when the mutated gene is neurologically expressed may overcome this limitation. For example, the deletion of a gene could be programmed to occur in the adult mouse after development is complete, thereby eliminating problems caused by developmental compensation. For more information about these techniques, see Sauer 1998.

The second factor is the mouse’s background genotype. Because many genes regulate complex behaviors and drug responses (Banbury Conference 1997), the expression of any one gene is influenced by the presence of the other genes in an organism’s genotype. Therefore, when a gene is knocked out in a particular inbred strain of mouse (i.e., populations of mice that are genetically identical), the background genotype is not necessarily silent and may mask differences in behavior because of the deleted gene. One way to control for this complication is to breed the deleted or mutated gene onto several inbred strain genetic backgrounds to assess the more complicated gene-gene interactions.

In addition, researchers must consider the possible confounding effects of the genetic background of the original ES cell used for homologous recombination. Some genetic material from the ES cell will be closely linked to the region of the mutated version of the gene. During recombination, when the altered gene replaces the wild-type gene, the ES cell’s genetic material will be carried with the mutated gene because of its close linkage. This association of ES cell DNA and the targeted gene will not be disrupted, even after several generations of breeding. If a change in behavior is observed in a gene-targeted mouse, the ES cell’s DNA, and not the altered gene, could be the cause. Therefore, when interpreting behavioral data, researchers should consider the phenotype of the inbred strain that is the source of the ES cell.

### Using Knockout and Knock-in Mice in Alcohol Research

Genes that encode receptor proteins are frequently selected as candidate genes in the gene-targeting approach. Several relevant receptor subunit knockout mouse lines have been used in alcohol research to evaluate contributions of a specific receptor subunit in alcohol’s behavioral actions, such as sedation, initial sensitivity, ataxia, withdrawal, tolerance, or consumption. For example, four lines of mice lacking the GABA_A_ receptor subunits α6, β3, γ2L, and δ have been specifically created to test for responses to alcohol and anesthetics (Bowers et al. 2000*b*; Mihalek et al 1999; Homanics et al. 1997, 1998, 1999; Quinlan et al. 1998). Surprisingly, mutant mice lacking α6, β3, and γ2L subunits failed to show different responses to alcohol compared with control mice, as measured by alcohol-induced sedation, tolerance, or withdrawal responses (i.e., α6 and γ2L) (Homanics et al. 1997, 1998, 1999). In addition, alcohol enhancement of GABA_A_ receptor inhibition, alcohol-induced reduction in anxiety, and alcohol-induced hyper-locomotion were not found to be different in γ2L mutant mice compared with control mice (Homanics et al. 1999). Although β3 mutant mice differed in their response to general anesthetics compared with nonmutant mice, they did not differ from the control mice in alcohol-induced sedation (Quinlan et al. 1998). Explanations for these unexpected results may involve some of the confounding factors previously discussed. For example, the lack of effects in the α6 knockout mice may be caused by interference from the mice’s genetic background or from overcompensation by other GABA_A_ subunits. The authors also suggest that the γ2S subunit may substitute for the missing γ2L subunit, masking a potential role of γ2L in alcohol sensitivity.

The amount of alcohol consumed in a free-choice drinking paradigm is one measure of the reinforcing effects of alcohol. Alcohol consumption was not evaluated in the GABA_A_ subunit knockout mice previously described; however, the δ knockout mice were tested for alcohol drinking. When δ mutant and wild-type mice were offered a choice between water and alcohol solutions ranging from 3 to 11 percent, mice lacking the δ subunit drank significantly less alcohol (Bowers et al. 2000*b*), suggesting that the δ subunit is one factor involved in alcohol preference. The results of drinking studies using several other receptor knockout mice, some of which are described later in this article, indicate that this behavior is multigenic.

Both the dopaminergic and serotonergic neurotransmitter systems have been implicated in alcohol-induced hyperlocomotion and the reinforcing effects of alcohol. Receptor proteins for each of these transmitters are derived from families of genes, several of which have been selected as gene-targeting candidates. The D_2_ dopamine receptor knockout mice were created based on human studies that implicated variants of the D_2_ receptor in alcoholism, although this association has not been found in every study (Goldman 1995). When D_2_ mutant and wild-type control mice were offered a choice of alcohol or water, the D_2_ mutant mice consumed significantly less alcohol (Phillips et al. 1998). A similar study of D_1_ receptor mutants also reported a decrease in consumption that may have been associated with higher levels of dopamine in some brain regions (El-Ghundi et al. 1998).

Decreased initial sensitivity has been associated with alcoholism in human populations (Schuckit 1988); therefore, this phenotype is frequently tested in mice using alcohol-induced increases in locomotor activity as the measure of sensitivity. Dopamine is thought to regulate basal as well as alcohol-induced hyperlocomotion. Contrary to human studies, however, the D_2_ knockout mice, which consumed less alcohol, also demonstrated reduced sensitivity to the activating effects of alcohol. On the other hand, mice lacking the D_4_ receptor were supersensitive to alcohol-induced locomotion; to date, these mice have not been tested for alcohol consumption (Rubinstein et al. 1997).

In contrast with the decrease in alcohol drinking observed in dopamine receptor knockout mice, initial studies of mice lacking the 5–HT_1B_ receptor showed that they consumed twice as much alcohol as wild-type control mice (Crabbe et al. 1996). However, the increased consumption demonstrated by the mutant mice was not replicated in later studies. This is most likely attributable to latent genetic background interactions between the ES cell genotype and the genotype of the inbred strain on which the mutation was bred. This interaction did not appear until after several generations of breeding had been conducted (Crabbe et al. 1999). Additional tests of alcohol-induced behaviors indicated that initial sensitivity to the ataxic effects of alcohol was also increased in 5–HT_1B_ mutants, whereas tolerance to chronic treatment developed more slowly and measures of withdrawal sensitivity were not affected by the deletion of the gene. These results are similar to those reported for the overexpressing 5–HT_3_ transgenics described earlier. The research conclusions indicating that both underexpressing and overexpressing 5–HT receptor mutants would have similar phenotypes may appear counterintuitive; however, the 5–HT receptors are heterogeneous and have different functions in the brain. For example, 5–HT_1B_ receptors regulate release of serotonin from the neuron, whereas activation of 5–HT_3_ receptors produces a rapid excitation of the neuron. Numerous other studies have reported the effect of single-gene mutations on alcohol drinking, indicating that many genes regulate this phenotype. (See the table for references to these and other receptor protein knockout studies.)

Alcohol research with knockout mice has also targeted certain enzyme proteins known as kinases. Protein kinases are enzymes that activate or deactivate the function of proteins, including receptor proteins, by attaching phosphate groups to the proteins. For example, protein kinase C (PKC) may modify, and thus affect the function of, the GABA_A_ receptor. The activation and deactivation of specific proteins are two components of a signaling mechanism through which chemical signals are relayed from the cell’s surface to its interior.

Research suggests that PKC function is associated with alcohol’s effects on the brain (Stubbs and Slater 1999). PKC is comprised of a family of enzyme subtypes (Parker 1994). Recently, knockout models of two of these subtypes have been tested for alcohol behaviors. PKCγ is exclusively expressed in the brain, including regions associated with alcohol sedation and ataxia. Tests of initial sensitivity to the sedating effects of alcohol demonstrated that mice lacking PKCγ were less sensitive than wild-type control mice. This may be related to PKCγ’s action at the GABA_A_ receptor, as alcohol-enhanced inhibition of the receptor function was absent in brain tissue from the null mutant mice compared with the wild-type controls (Harris et al. 1995). In addition, rapid and chronic tolerance to alcohol was decreased in these mice, albeit dependent on background genotype (Bowers et al. 1999, 2000*a*). PKCɛ is also highly expressed in the brain and is localized in some but not all of the same brain regions as PKCγ. Tests of alcohol consumption have indicated that mice lacking PKCɛ increased their drinking 200 percent over that of control mice and, in contrast with PKCγ mutants, demonstrated an *increased* sensitivity to alcohol-induced sedation (Hodge et al. 1999).

Tests of alcohol sensitivity in kinase knockout mice are not limited to the PKC family of enzymes. Recently, research has been conducted using the following types of mice: mice lacking the protein kinase A regulatory subunit βII (i.e., PKAβII), mice lacking the tyrosine kinase Fyn (i.e., a kinase of the amino acid tyrosine), dopamine beta hydroxylase knockout mice (i.e., mice lacking the dopamine beta form of the hydroxylase enzyme), and mice lacking forms of the gene for aldehyde dehydrogenase (i.e., an enzyme involved in alcohol metabolism) (see table).

From these investigations of knockout and transgenic mice, researchers can easily appreciate the complexity of alcohol’s actions and the difficulty in definitively interpreting the contributions of individual proteins to alcoholism. The examples discussed in this article are by no means an exhaustive representation of all mutant mouse models used in alcohol research (see table). The techniques of reverse genetics continually evolve, and as new models are developed, scientific and clinical understanding of the neurobiology of alcoholism will most certainly continue to advance.

## Figures and Tables

**Table:** Transgenic and Knockout Mouse Models Used in Alcohol Research

Candidate Gene/Protein	Phenotype	Authors	Reference
**Transgenic models**			
Acet aldehyde (ADH) *and*	Exacerbation of alcoholic heart muscle disease in transgenic hearts	Liang, Q., et al.	*Journal of Pharmacology Experimental Therapeutics* 291:766–772, 1999
Adenylate cyclase 7 (AC7)	Reduced initial sensitivity to alcohol-induced ataxia and sedation in transgenic mice	Yoshimura, M., et al.	*Alcoholism: Clinical and Experimental Research* 23:92A, 1999
*Bcl-2* (cell death ) repressor gene	Overexpression of *bcl-2* protects cerebellar neurons from alcohol neurotoxicity	Heaton, M.B., et al.	*Brain Research* 817:13–18, 1999
Gama-aminobutyric acid_A_ (GABA_A_) γ2L and γ2S subunits	Decreased acute functional tolerance in both transgenic lines, but no differences in alcohol-induced sedation or acute withdrawal or alcohol potentiation of GABAergic function	Wick, M.J., et al.	*European Journal of Neuroscience* in press
5–HT_3_ receptor	Decreased alcohol consumption in transgenics	Engel, S.R., et al.	*Psychopharmacology* 140:243–248,1998
	Enhanced sensitivity to alcohol-induced hyperlocomotion, but no difference in sedation	Engel, S.R., et al.	*Psychopharmacology* 144:411–415, 1999
HIV-1 transactivator protein (HIV-1 Tat)	Alcohol exposure enhances the progression of certain HIV-1 disease traits in transgenic mice	Prakash, O., et al.	*Alcoholism: Clinical and Experimental Research* 22:266S–268S, 1998
Human transforming growth factor α (TGFα)	Aggression is *not* reduced after alcohol in highly aggressive transgenic male mice	Hilakivi-Clarke, L., et al.	*Neuroreport* 4:155–158, 1993
Neuropeptide Y (NPY)	Decreased alcohol consumption in transgenics and increased sensitivity to alcohol sedation	Thiele, T.E., et al.	*Nature* 396:366–369, 1998
**Knockout models**			
Aldehyde dehydrogenase (ADH) classes 1, 3 and 4	ADH 1 mutant mice: increased sensitivity to sedative effects; ADH 1 and 4: reductions in blood alcohol clearance in mutant mice	Deltour, L., et al.	*Journal of Biochemistry* 274:16796–16801, 1999
Cytochrome P_450_ (CYP)2E1	Alcohol elimination rate unchanged and no effect of the null mutation on alcohol-induced liver injury	Kono, H., et al.	*American Journal of Physiology* 277:G1259–G1267, 1999
Dopamine (DA) D_1_ receptor	Decreased alcohol consumption in mutant mice	El-Ghundi, M., et al.	*European Journal of Pharmacology* 353:149–158, 1998
DA_2_ receptor	Decreased alcohol consumption and reduced sensitivity to alcohol-induced locomotor impairment in null mutant mice	Phillips, T.J., et al.	*Nature Neuroscience* 1:610–615, 1998
DA_4_ receptor	Knockout mice display increased sensitivity to alcohol-induced locomotor activity	Rubenstein, M., et al.	*Cell* 90:991–1001, 1997
Dopamine β hydroxylase	Knockout results in norepinephrine depletion; mutants display decreased alcohol preference and increased sensitivity to alcohol-induced sedation and hypothermia	Weinshenker, D., et al.	*Journal of Neuroscience* 20:3157–3164, 2000
Fyn kinase	Mutants exhibit increased sensitivity to sedative effects of alcohol and a lack of alcohol-enhanced phosphorylation of *N*-methyl-d-aspartate (NMDA) receptors	Miyakawa, T., et al.	*Science* 278:698–701, 1997
GABA_A_ α6 receptor subunit	No effect of null mutation on alcohol sedation	Homanics, G.E., et al.	*Molecular Pharmacology* 51:588–596, 1997
	No effect of null mutation on acute or chronic alcohol tolerance or withdrawal hyperexcitability	Homanics, G.E., et al.	*Alcoholism: Clinical and Experimental Research* 22:259–265, 1998
GABA_A_ β3 receptor subunit	Knockout mice do not differ from controls in alcohol-induced sedation	Quinlan, J.J., et al.	*Anesthesiology* 88:775–780, 1998
GABA_A_ γ2L subunit	Mutant mice display normal responses to alcohol potentiation of GABAergic receptor function, sedation, anxiolysis, AFT, withdrawal seizures, and hyperlocomotor activity	Homanics, G.E., et al.	*Neuropharmacology* 38:253–265, 1999
GABA_A_ δ receptor subunit	Null mutants consume less alcohol than controls	Bowers, B.J., et al.	*Alcoholism: Clinical and Experimental Research* 24:97A, 2000
Neuropeptide Y (NPY)	Knockout mice consume more alcohol than controls and are less sensitive to alcohol sedation	Thiele, T.E., et al.	*Nature* 396:366–369, 1998
NPY Y5 receptor	No effect of null mutation on alcohol consumption	Thiele, T.E., et al.	A*lcoholism: Clinical and Experimental Research 23:61A, 1999*
Protein kinase A (PKA) regulatory II β (βII) subunit	Increased alcohol consumption and decreased initial sensitivity to sedation in knockout mice	Thiele, T.E., et al.	*Journal of Neuroscience* 20:RC75, 2000
Protein kinase C γ subunit (PKCγ)	Decreased development of rapid and chronic tolerance to sedation and hypothermia in mutant mice but dependent on genetic background Decreased initial sensitivity to sedation, hypothermia, and alcohol potentiation of GABA_A_ receptor function	Bowers, B.J., et al.	*Alcoholism: Clinical and Experimental Research* 23:387–397, 1999
		Bowers, B.J., et al.	*Addiction Biology* 5:47–58, 2000
		Harris, R.A., et al.	*Proceedings of the National Academy of Sciences* 92:3658–3662, 1995
Protein kinase C ɛ subunit (PKCɛ)	Null mutant mice exhibit increased sensitivity to alcohol sedation and hyperlocomotion and alcohol potentiation of GABA_A_ receptor function Decreased alcohol consumption	Hodge, C.W., et al.	*Nature Neuroscience* 2:997–1002, 1999
Serotonin 1B (5-HT_1B_) receptor	Elevated alcohol consumption in null mutants, but see text (Crabbe et al., *Science* 284:1670–1671, 1999)	Crabbe, J.C., et al.	*Nature Genetics* 14:98–101, 1996
	Lack of alcohol-induced conditioned place preference in knockouts; normal alcohol-induced taste aversion	Risinger, F.O., et al.	*Alcoholism: Clinical and Experimental Research* 20:1401–1405, 1996
	Modest increase in rate of responding for alcohol in operant paradigm by knockout mice	Risinger, F.O., et al.	*Behavioral Brain Research* 102: 211–215, 1999
Vesicular monoamine transporter 2 (VMAT2)	Lethal in homozygous mutants; increased alcohol-induced hyperlocomotion in heterozygotes	Wang, Y-M., et al.	*Neuron* 19:1285–1295, 1997

## Footnotes

1 The source of this gene could be mouse or another mammalian species, including human.

2 Homologous recombination refers to the exchange of genetic material between complimentary fragments of DNA.
